# Dipole orientation analysis without optical simulation: application to thermally activated delayed fluorescence emitters doped in host matrix

**DOI:** 10.1038/s41598-017-08708-1

**Published:** 2017-08-16

**Authors:** Takeshi Komino, Yuji Oki, Chihaya Adachi

**Affiliations:** 1 Education Center for Global Leaders in Molecular System for Devices, Kyushu University, 744 Motooka, Nishi, Fukuoka 819-0395 Japan; 2 Center for Organic Photonics and Electronics Research (OPERA), Kyushu University, 744 Motooka, Nishi, Fukuoka 819-0395 Japan; 3 Japan Science and Technology Agency, ERATO, Adachi Molecular Exciton Engineering Project, 744 Motooka, Nishi, Fukuoka 819-0395 Japan; 4 Department of Electronics, Kyushu University, 744 Motooka, Nishi, Fukuoka 819-0395 Japan; 5 International Institute for Carbon Neutral Energy Research (WPI-I2CNER), Kyushu University, 744 Motooka, Nishi, Fukuoka 819-0395 Japan

## Abstract

The dipole orientation of guest emitters doped into host matrices is usually investigated by angular dependent photoluminescence (PL) measurements, which acquire an out-of-plane PL radiation pattern of the guest-host thin films. The PL radiation patterns generated by these methods are typically analysed by optical simulations, which require expertise to perform and interpret in the simulation. In this paper, we developed a method to calculate an orientational order parameter *S* without the use of full optical simulations. The PL radiation pattern showed a peak intensity (*I*_sp_) in the emission direction tilted by 40°–60° from the normal of the thin film surface plane, indicating an inherent dipole orientation of the emitter. Thus, we directly correlated *I*_sp_ with *S*. The *S* − *I*_sp_ relation was found to depend on the film thickness (*d*) and refractive indices of the substrate (*n*_sub_) and the organic thin film (*n*_org_). Hence, *S* can be simply calculated with information of *I*_sp_, *d*, *n*_sub_, and *n*_org_. We applied our method to thermally activated delayed fluorescence materials, which are known to be highly efficient electroluminescence emitters. We evaluated *S* and found that the error of this method, compared with an optical simulation, was less than 0.05.

## Introduction

In the last decade, research into organic light-emitting diodes (OLEDs) has developed remarkably, supported by advances in control over molecular dipole orientation in amorphous films^1^. In thin glassy films used as OLED materials dipoles horizontally oriented to the substrate surface can greatly improve device performance by increasing the outcoupling efficiency of electroluminescence (EL)^2–4^. Light-emitting layers are generally guest-host binary molecular systems, hence control is needed over the orientation of the emissive dye (guest molecule) embedded into the host matrix. Some recent studies have demonstrated an outstanding external EL quantum efficiency (EQE) of over 30% by engineering a horizontal dipole orientation of thermally activated delayed fluorescence (TADF) emitters^5–7^. This class of emitters can harvest triplet excitons to attain high EQE values equivalent to those of phosphorescence emitters^8–10^. Angular dependent photoluminescence (PL) measurements, developed by Brütting and co-workers, are the most effective method of assessing dipole orientation in thin films^2, 4^. This method can be used to measure the out-of-plane PL intensity distribution of guest-host thin films, hereafter referred to as a radiation pattern. The radiation pattern is analysed by simulating the pattern that would result from various dipole orientations. Thus, one can determine the dipole orientation that reproduces the experimentally obtained radiation pattern. The dipole orientation is a fundamental property of glassy and soft organic materials which governs their photonic, electronic, excitonic, and optical characteristics; hence this method is applicable not only to OLEDs but many other research fields. However, this method requires expertise in optical simulation to analyse the radiation patterns and not all researchers are able to easily determine dipole orientations. A simple relation that directly connects the radiation pattern to the dipole orientation would enable more ready evaluation of dipole orientations without full optical simulations. This would allow wider uptake and application of this useful technique.

To date, four expression have been proposed to define dipole orientation as follows. (i) The ratio of *p*_x_, *p*_y_, and *p*_z_, where *p* is the existence probability of a dipole^2^. The subscripts x, y, and z denote the direction of Cartesian coordinates where x and y are parallel to the substrate plane and z is orthogonal to the x-y plane. For a uniaxial dipole orientation, *p*_x_ = *p*_y _≠ *p*_z_. (ii) The ratio of vertically oriented dipoles versus the total dipoles is given by *Θ*_v_ = *p*_z_/(*p*_x_ + *p*_y_ + *p*_z_)^11^. The dipoles are also occasionally substituted by the radiation power in this relation^12^. (iii) The ratio of horizontally oriented dipoles versus the total dipole is given by *Θ*_h_ = (*p*_x_ + *p*_y_)/(*p*_x_ + *p*_y_ + *p*_z_)^6, 13^. (iv) An orientational order parameter can be calculated as *S* = (*p*_z_^2^ − *p*_x_^2^)/(*p*_z_^2^ + 2 *p*_x_^2^)^14^. Any of these parameters may be arbitrarily selected for analysis of the dipole orientation. In our previous studies, we have selected *S* for two reasons. First, *S* can express the uniaxial orientational order considering the distribution of dipole orientations in a system (e.g., crystals or glasses). Second, *S* values in guest-host systems can be compared with those in neat films; molecular orientations in neat films have been evaluated by *S*, so far. Note that *S* is defined as the order parameter for the emission ability (*p*_z_^2^ and *p*_x_^2^) here, rather than that for the molecular dipoles themselves (*p*_z_ and *p*_x_). In this paper, we sought a relation, to directly connect experimentally obtained radiation patterns with *S*. We found that *S* can be directly determined from the second peak intensity in the radiation pattern without the use of a full optical simulation.

## Results

First, we briefly describe the experimental method used to acquire radiation patterns. A detailed procedure is described elsewhere^14^. An approximately 15-nm-thick guest-host film is deposited on a transparent substrate (e.g., non-fluorescent glass or fused silica) and then encapsulated with a cover slip. Use of such an ultra-thin film enables us to assume the uniform exciton generation by photoexcitation, resulting in the simple optical model for the analysis. The substrate is attached to the flat plane of a fused silica half cylinder prism with matching oil having a refractive index close to that of the substrate. The thin film sample is subjected to an excitation light beam and the photoluminescence intensity in transverse magnetic (TM) mode is detected radially from the half cylinder prism. The radiation pattern is obtained by plotting the monochromatic PL intensity as a function of angle (emission angle, *θ*) between the normal of the substrate (*θ* = 0°) and the emission direction in the x-z plane.

**Figure 1:**
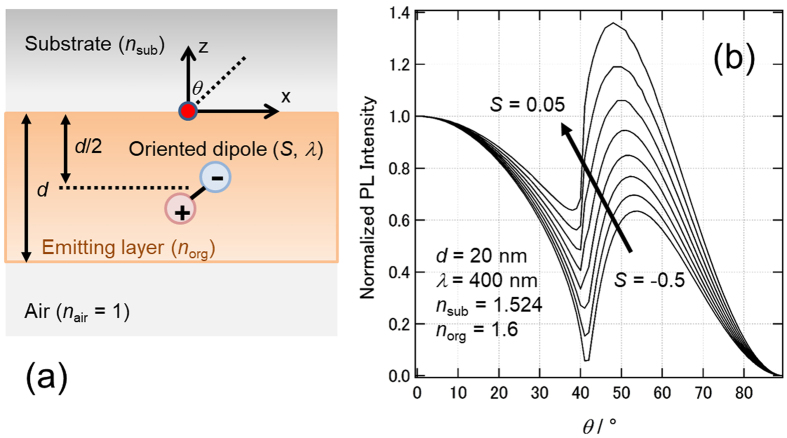
(**a**) Schematic model of the photonic mode simulation. (**b**) Simulation result for *d* = 20 nm, *λ* = 400 nm, *n*_sub_ = 1.524, and *n*_org_ = 1.6.

To determine a connection between the radiation pattern and *S*, we performed an optical simulation. A schematic model of the optical simulation is depicted in Fig. 1a. We set *S*_sim_ as the main variable, where the subscript “sim” indicates that the value was obtained by simulation. The radiation patterns were compared with simulated results with various values of *S*_sim_. Figure 1b shows an example of the resulting radiation pattern when *d* = 20 nm, *λ* = 400 nm, *n*_sub_ = 1.524, and *n*_org_ = 1.6 were used (light intensity was normalized at 0°). These curves closely matched the experimentally obtained radiation patterns. We aimed to identify any special features of the curves that directly correlated with *S*. Looking at trends of the curves, we recognized that the second peak intensity (*I*_sp_) at 40°–60° monotonically increased as *S*_sim_ increased. Accordingly, we selected *I*_sp_ as a parameter to characterize the curves, where the parameters governing *S*_sim_ are *I*_sp_, *d*, *λ*, *n*_sub_, *n*_air_ and *n*_org_. To identify a general way to correlate these values, we applied the following strategy: (i) the parameter *ξ* is defined by the values of *λ*, *n*_air_ and *n*_org_. (ii) We considered a three-dimensional space defined by *S*_sim_, *I*_sp_, and *ξ*. The definition of *ξ* was modified so that the simulated plots featured a smooth surface in *S*_sim_ − *I*_sp_ − *ξ* space. (iii) For various values of *d* and *n*_sub_ we found an equation describing the smooth surface, which correlated *S*_sim_ with *I*_sp_ and *ξ*. We used this equation to define the calculated *S* (*S*_calc_). For practical purposes, *S*_calc_ can be calculated from the equation from the experimentally obtained *I*_sp_ and a parameter set, which is defined by *d*, *λ*, *n*_sub_, *n*_air_, and *n*_org_.

**Figure 2:**
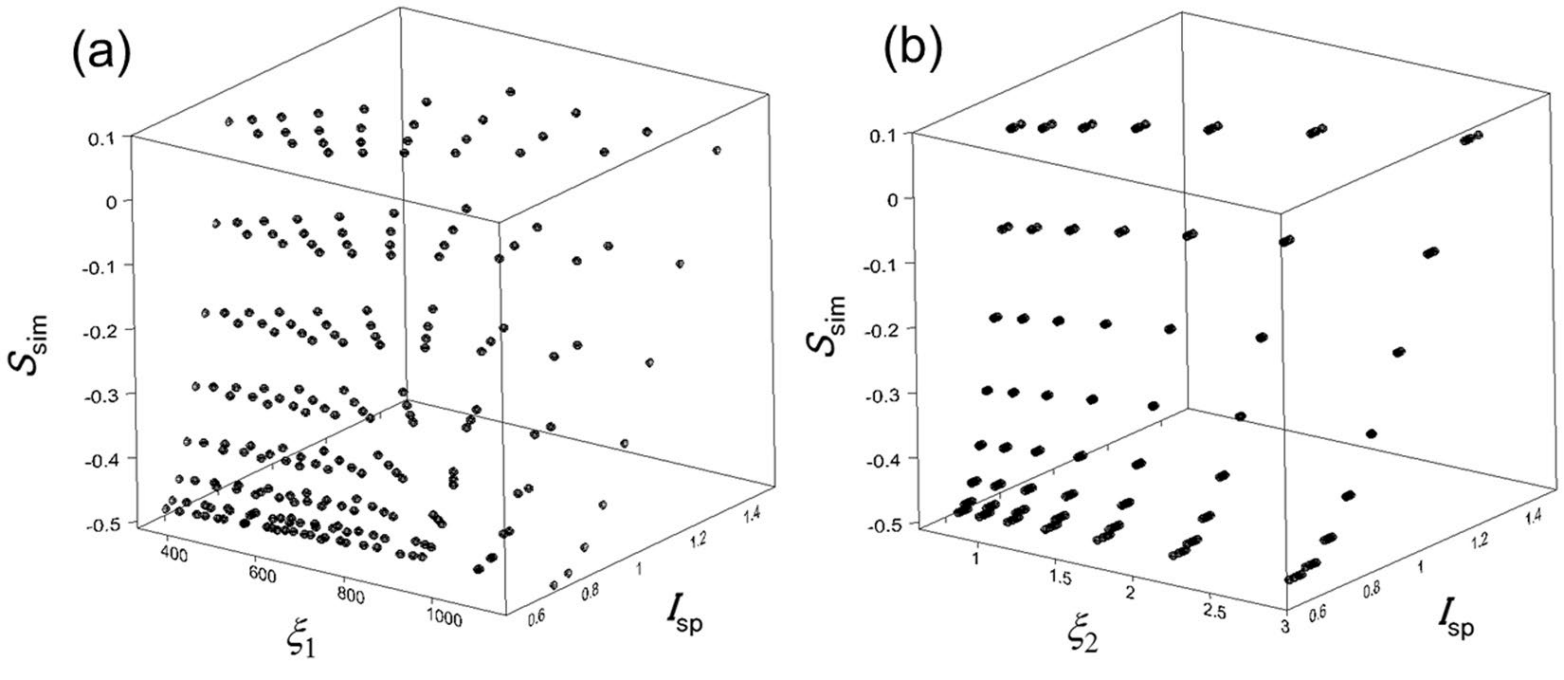
Simulated results of *S*_sim_ obtained by *I*_sp_ and (**a**) *ξ*_1_ or (**b**) *ξ*_2_.

In step (i), to find an appropriate *ξ*, which created a smooth surface in *S*_sim_ − *I*_sp_ − *ξ* space, we arbitrarily considered the formula *ξ*_1_ = *λ*/(*n*_org_ − *n*_air_) as an initial example. We defined *ξ*_1_, in this way because it is plausible that the beam propagation may correlate with a wave vector. Figure 2a shows scatter plots in the *S*_sim_ − *I*_sp_ − *ξ*_1_ space, which were obtained from a simulation with *d* = 20 nm and *n*_sub_ = 1.524. We set *S*_sim_ as the z axis, because *S*_calc_ becomes the desired calculation result in the practical use of the proposed method. As shown in Fig. 2a, *ξ*_1_ did not converge to create a smooth, indicating that *ξ*_1_ is not an appropriate parameter for calculation of *S*_calc_. Thus, we modified the definition of *ξ* to achieve well-organized scattered plots as step (ii). When we tested a new parameter *ξ*_2_, the scattered plots were well organized as shown in Fig. 2b. *ξ*_2_ is given by equation 1,

1 $${\xi }_{2}={[({n}_{org}-{n}_{air})]}^{-2}.$$

The plots in *S*_sim_ − *I*_sp_ − *ξ*_2_ space were insensitive to variation of *λ*. Four dots for 400, 500, 600, and 700 nm appeared at almost the same coordinates in Fig. 2b. This was because *d* is much smaller than *λ* and the effect of *λ* on the *S*_sim_ − *I*_sp_ − *ξ*_2_ plots was negligible unless a much thicker film (*d* ≫ 20 nm) was used.

**Figure 3:**
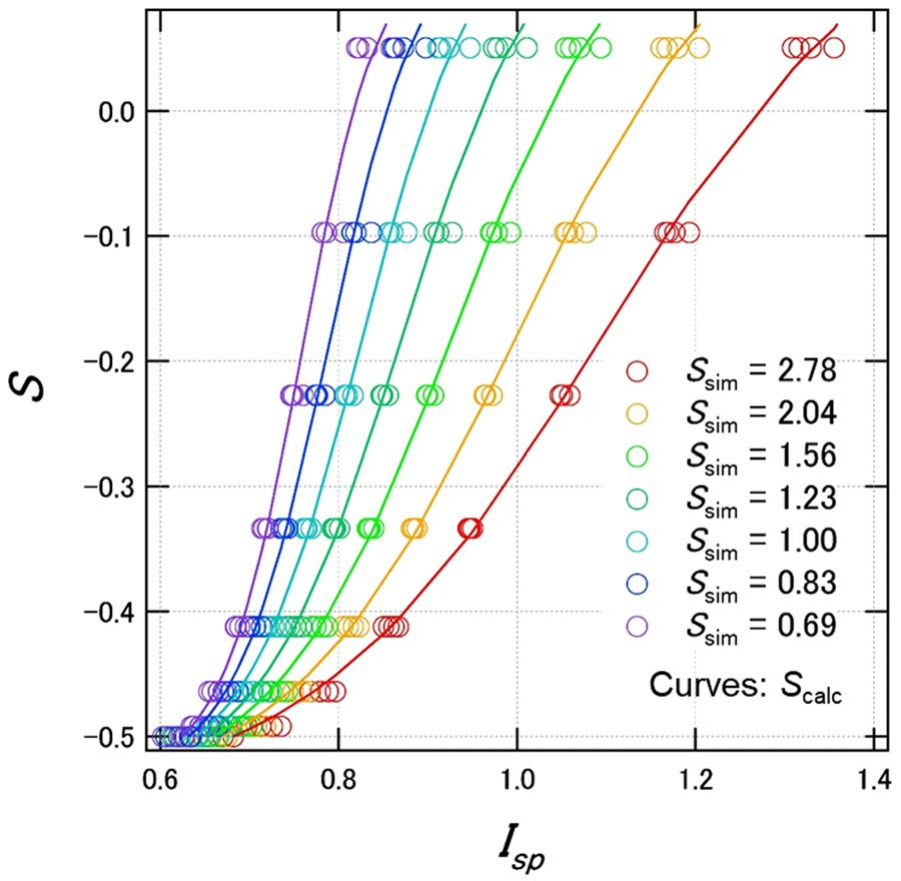
*S* − *I*_sp_ characteristics at *d* = 20 nm and *n*_sub_ = 1.524. Dots indicate *S*_sim_, i.e., a representation of the *S*_sim_ − *I*_sp_ − *ξ*_2_ plots (Fig. 2b) viewed from the *S* − *I*_sp_ plane. The sigmoidal curves show fitting results of *S*_calc_ (eg. 1).

**Figure 4:**
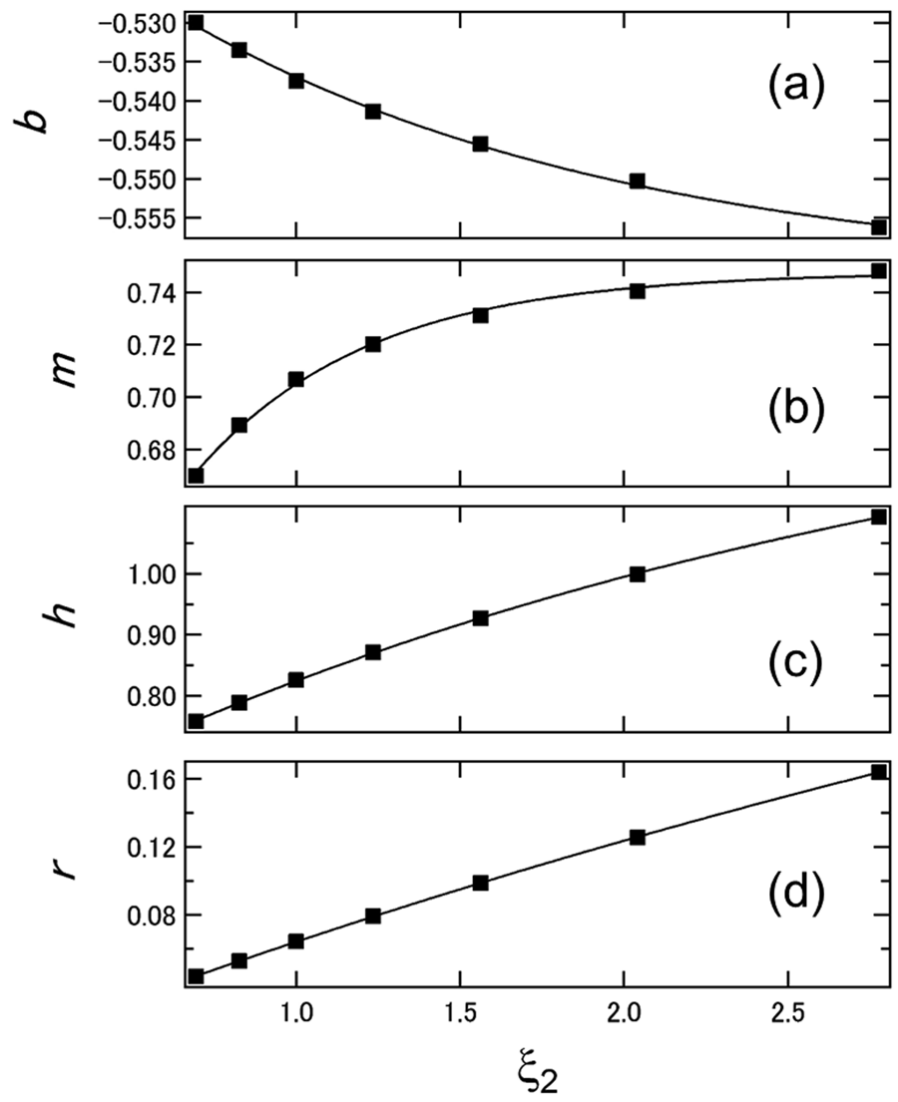
(**a**) *b*, (**b**) *m* (**c**) *h*, and (**d**) *r* as a function of *ξ*_2_. The characteristics were obtained at *d* = 20 nm and *n*_sub_ = 1.524.

**Figure 5:**
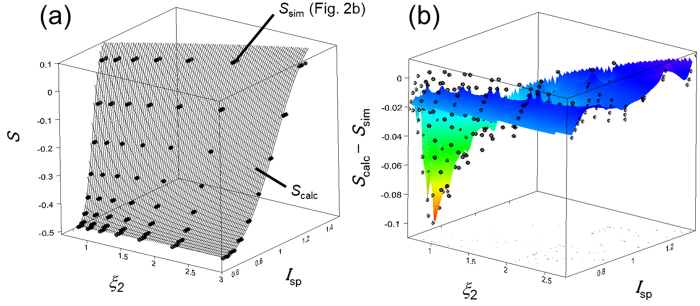
(**a**) *S*_calc_ − *I*_sp_ − *ξ*_2_ (surface) and *S*_sim_ − *I*_sp_ − *ξ*_2_ (dots) characteristics from the conditions *d* = 20 nm and *n*_sub_ = 1.524. The *S*_calc_ surface closely replicated *S*_sim_, suggesting that the *S*_calc_ − *I*_sp_ − *ξ*_2_ characteristics can be used to calculate *S*_calc_ at an arbitrary condition defined by *I*_sp_, *d*, *λ*, *n*_sub_, *n*_air_ and *n*_org_; the substrate should be glass or fused silica. (**b**) Error between *S*_calc_ and *S*_sim_ for each value of *ξ*_2_ and *I*_sp_ at *d* = 20 nm and *n*_sub_ = 1.524 (see Table 1).

Next, in step (iii), we derived an equation expressing the *S*_calc_ surface. To simplify the derivation, we first correlated the plots in the *S*_sim_ − *I*_sp_ plane, and then extended these to correlate the *S*_sim_ − *I*_sp_ relation with *ξ*_2_. Figure 3 shows the *S*_sim_ − *I*_sp_ characteristics, which represent the *S*_sim_ − *I*_sp_ − *ξ*_2_ plots (Fig. 2b) viewed from the *S*_sim_ − *I*_sp_ plane. Each curve showed a sigmoid-like curve. Thus, we fitted the plots with a sigmoidal function, given by equation 2:

2 $${S}_{calc,{I}_{sp},{\xi }_{2},d,{n}_{sub}}={b}_{{\xi }_{2},d,{n}_{sub}}+\frac{{m}_{{\xi }_{2},d,{n}_{sub}}}{1+exp(\{{h}_{{\xi }_{2},d,{n}_{sub}}-{I}_{sp}\}/{r}_{{\xi }_{2},d,{n}_{sub}})},$$

where, *b* is the baseline of the *S*_calc_ − *I*_sp_ characteristics, *m* is the maximum value of the sigmoidal curve, *h* is the specific *I*_sp_ value when *S*_calc_ = *h*/2, and *r* is the inverse rate of increase of *S*_calc_ versus *I*_sp_. Each plot of *S*_sim_ was reproduced well by eq. 2, demonstrating that the *S*_calc_ − *I*_sp_ relation could form the basis of an equation to reproduce the *S*_sim_ − *I*_sp_ − *ξ*_2_ surface. Next, we investigated the correlation of *b*, *m*, *h*, and *r* with *ξ*_2_. The purpose of this operation was to correlate the *S*_calc_ − *I*_sp_ relation with *ξ*_2_; this is equivalent to looking down the three-dimensional *S*_sim_ − *I*_sp_ − *ξ*_2_ space from the *I*_sp_ − *ξ*_2_ plane (Fig. 2b). Figures 4a–d show the dependences of *b*, *m*, *h*, and *r* on *ξ*_2_. The variables *b*, *m*, *h*, and *r* all exhibited clear dependences on *ξ*_2_. The shapes of the curves suggested an exponential function, hence we fitted these with equations 3–6,

3 $${b}_{{\xi }_{2},d,{n}_{sub}}={a}_{d,{n}_{sub}}exp[-{k}_{d,{n}_{sub}}\{{\xi }_{2}-{c}_{d,{n}_{sub}}\}]+{e}_{d,{n}_{sub}},$$

4 $${m}_{{\xi }_{2},d,{n}_{sub}}={a}_{d,{n}_{sub}}^{{}^{{\prime} }}(1-exp[-{k}_{d,{n}_{sub}}^{{}^{{\prime} }}\{{\xi }_{2}-{c}_{d,{n}_{sub}}^{{}^{{\prime} }}\}])+{e}_{d,{n}_{sub}}^{{}^{{\prime} }},$$

5 $${h}_{{\xi }_{2},d,{n}_{sub}}={a}_{d,{n}_{sub}}^{{\prime}{\prime}}(1-exp[-{k}_{d,{n}_{sub}}^{{\prime}{\prime}}\{{\xi }_{2}-{c}_{d,{n}_{sub}}^{{\prime} {\prime}}\}])+{e}_{d,{n}_{sub}}^{{\prime}{\prime}},$$

6 $${r}_{{\xi }_{2},d,{n}_{sub}}={a}_{d,{n}_{sub}}^{{{\prime}{\prime}}{{\prime} }}(1-exp[-{k}_{d,{n}_{sub}}^{{{\prime}{\prime}}{{\prime}}}\{{\xi }_{2}-{c}_{d,{n}_{sub}}^{{\prime}{\prime}{\prime}}\}])+{e}_{d,{n}_{sub}}^{{\prime}{\prime} {\prime}},$$

where, *a*, *a*′, *a*″, *a*″′, *k*, *k*′, *k*″, *k*″′, *c*, *c*′, *c*″, *c*″′, *e*, *e*′, *e*″′, and *e*″ are constants characterizing the *b* − *ξ*_2_, *m* − *ξ*_2_, *h* − *ξ*_2_, and *r* − *ξ*_2_ curves. As shown in Fig. 4a–d, good fits were obtained with eqs 3–6. Hence the *S* values were arbitrarily calculated with eqs 2–6 based on these parameters. Table 1 summarizes all the parameters that we used to calculate the *S*_calc_ − *I*_sp_ − *ξ*_2_ surface, as shown in Fig. 5a. Here, we used *d* = 20 nm and *n*_sub_ = 1.524 (glass substrate). A surface plot was generated over the scattered *S*_sim_ − *I*_sp_ − *ξ*_2_ dots (Fig. 2b) to allow comparison. As shown in Fig. 5a, the calculated surface closely followed the simulated dots. To verify the reproducibility of our calculation compared with optical simulations, *S*_calc_ − *S*_sim_ was evaluated. This result is shown in Fig. 5b. The interpolated surface in the figure was generated by the spline approximation. In the ranges of 0.69 ≤ *ξ*_2_  ≤ 2.78 and 0.618 ≤ *I*_sp_  ≤ 1.32, the error was less than 0.111 at (*ξ*_2_, *I*_sp_) = (0.69, 0.746). Furthermore, in the typical range of experimental conditions 1.6 ≤ *n*_org_  ≤ 2.0, which corresponds to 1.99 ≤ *ξ*_2_  ≤ 2.78, the error was less than 0.040. The error comes mainly from the scattered *S*_sim_ values, which depend on *λ*. As mentioned earlier, the effect of *λ* on the *S*_sim_ − *I*_sp_ − *ξ*_2_ plots was negligible, but only small scattering was included. Here, it should be noted that the scale of the error can be considered very small for the practical use. Thus, we conclude that our method is of potential use in the direct calculation of *S*_calc_ from experimental angular dependent PL measurements.

**Table 1:** All parameters for *S*_calc_ and their error from *S*_sim_.

Substrate	Glass (*n*_sub_ = 1.524)			Fused silica (*n*_sub_ = 1.460)		
*d*/nm	10	15	20	10	15	20
*a*	0.098604	0.110944	0.042013	0.197569	0.156029	0.11373
*k*	0.690732	0.178521	0.736613	0.96795	0.792135	0.397617
*c*	−1.18994	−2.72364	0.345428	−1.21634	−1.50049	−1.82734
*e*	−0.56855	−0.601617	−0.56287	−0.567663	−0.565258	−0.575086
*a*′	0.366794	0.36543	0.359072	0.372707	0.367789	0.360643
*k*′	17.1356	0.275624	1.90403	4.92218	2.34445	1.40428
*c*′	0.543513	−10.8917	−0.117778	0.228131	−0.464428	−0.358297
*e*′	0.400497	0.405369	0.388698	0.397003	0.394126	0.39185
*a*″	0.644241	0.706328	0.689067	0.597502	0.618666	0.679183
*k*″	0.356334	0.307937	0.345435	0.334855	0.330799	0.298034
*c*″	0.441737	0.582837	0.526038	0.702383	0.737254	0.76181
*e*″	0.72579	0.746449	0.720144	0.722417	0.719095	0.719382
*a*″′	0.328729	0.192264	0.393989	0.194786	0.221476	0.2802
*k*″′	0.177307	0.165193	0.159079	0.159687	0.147584	0.125966
*c*″′	1.23618	5.02439	1.17711	5.11488	5.16589	5.29678
*e*″′	0.071262	0.243959	0.0754636	0.236137	0.248144	0.264477
Max. error of *S*_calc_	0.076^a^	0.048^a^	0.111^a^	0.067^a^	0.034^*a*^	0.064^*a*^
	(0.021)^b^	(0.020)^b^	(0.040)^b^	(0.020)^b^	(0.016)^*b*^	(0.039)^*b*^

^a^1.6 ≤ *n*_org_  ≤ 2.2, ^b^1.6 ≤ *n*_org_ ≤ 2.0.

**Figure 6:**
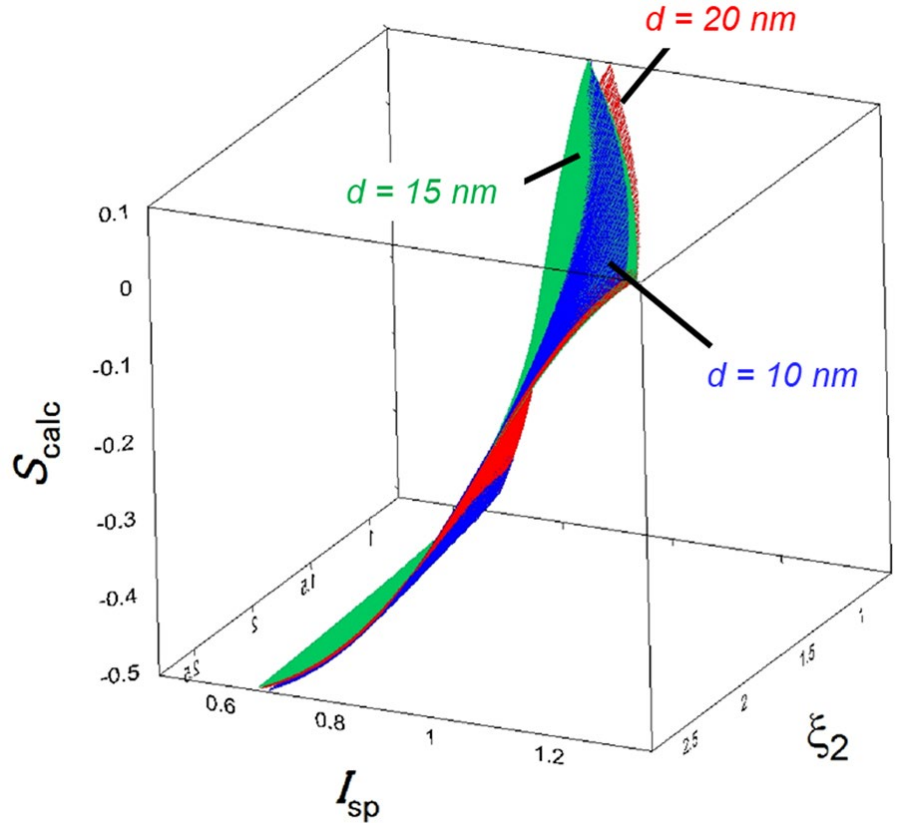
Variation of the *S*_calc_ − *I*_sp_ − *ξ*_2_ surfaces for *d* = 10, 15, and 20 nm, calculated with *n*_sub_ = 1.524 (for a glass substrate).

We could develop the method for calculating *S*_calc_ from the parameters *ξ*_2_ and *I*_sp_. However, here we should note that the parameters *a*, *a*′, *a*″, *a*″′, *k*, *k*′, *k*″, *k*″′, *c*, *c*′, *c*″, *c*″′, *e*, *e*′, *e*″, and *e*″′ were dependent on *n*_sub_ and *d*, meaning that the scope of this calculation method is limited by *n*_sub_ and *d*. However, both *n*_sub_ and *d* are predetermined by the sample preparation. Therefore, this limitation does not greatly influence the practical applicability of this approach to calculations. Glass (*n*_sub_ = 1.524) and fused silica (*n*_sub_ = 1.460) substrates are often used in angular dependent PL measurements, hence parameters for both substrates are listed in Table 1. Owing to the variation of *d*, the parameters for *d* = 10, 15, 20 nm are also independently listed in Table 1. One would consider whether the dipole orientation in such an ultra-thin film can be preserved in much thicker films. This is the case that one should carefully estimate the difference in the dipole orientations between the ultra-thin and thick films, especially for films as thick as 100 nm. Meanwhile, in the case of several tens of nm, it has been reported so far that the estimated orientation can be utilized to expect EL light outcoupling^7, 14, 15^. Thus, even in ultra-thin films, the estimated orientation can be used for considering the optical effect in practical devices. As shown in Fig. 6, the shape of the *S*_calc_ surface depends on *d*. The differences among the *S*_calc_ surfaces was very small, but significant. From a technical viewpoint, reliably adjusting the value of *d* on a nanometre scale is difficult to achieve experimentally (e.g., through vacuum vapour deposition). If *d* is not defined with sufficient accuracy, its value may be confirmed by an annealing treatment of the film above a temperature where the molecular configuration of the host matrix becomes disordered (this temperature often coincides with the material’s glass transition temperature)^7^. After annealing, *S*_calc_ should become zero. Thus, one can estimate *d*, and determine the appropriate *S*_calc_ surface from the crossing point of the experimentally obtained *I*_sp_ and *ξ*_2_ where *S*_calc_ = 0.

To test the validity of the calculation method, we calculated *S* values based on previous publications and compared our *S*_calc_ with the reported *S* values, which were obtained by photonic mode density simulations. This validation is equivalent to a comparison between our calculation method of *S*_calc_ and the optical simulation. We selected TADF emitters doped into conventional host matrices, because TADF molecules have been reported to show a wide range of *S* values from −0.50 to 0.05. The binary molecular systems used were 10-[4-(4,6-diphenyl-1,3,5-triazin-2-yl)phenyl]-10*H*-phenoxazine (PXZ-TRZ): 3,3-di(9*H*-carbazol-9-yl)biphenyl (mCBP), 2,6-bis(4-(10*H*phenoxazin-10-yl)phenyl)benzo[1,2-*d*:5,4-*d*ʹ] bis(oxazole) (*cis-*BOX 2): 4,4ʹ -bis(*N*-carbazolyl)-1,1′-biphenyl (CBP), and 9-[4-(4,6-diphenyl-1,3,5-triazin-2-yl)phenyl]-*N*3,*N*3,*N*6,*N*6-tetraphenyl-9*H*-carbazole-3,6-diamine (DACT-II):CBP. Table 2 summarizes the *S*_calc_ and reported *S* values. Even for the largest error, the differences between the calculated and simulated values were less than 0.05, and in good agreement with our estimation (Fig. 5b).

**Table 2:** Comparison of *S*_calc_ and reported *S* values.

Sample	*λ*/*nm*	*d*/*nm*	*n*_sub_	*n*_org_	*S*_calc_	*S* (reported)	Difference	Reference
PXZ-TRZ:mCBP^a^	530	15	1.524	1.74	0.048	0.05	0.002	14
PXZ-TRZ:mCBP^b^	530			1.74	−0.142	−0.12	0.022	14
PXZ-TRZ:mCBP^c^	530			1.73	−0.357	−0.31	0.047	14
*Cis-*BOx2:CBP^a^	510			1.76	−0.452	−0.41	0.042	7
*Cis-*BOx2:CBP^c^	510			1.75	−0.518	−0.5	0.018	7
DACT-II:CBP	515		1.46	1.76	−0.339	−0.29	0.049	15

Vapour deposition at ^a^300 K, ^b^250 K, and ^c^200 K.

While the dipole orientation of a guest emitter doped in a host matrix has been evaluated by a combination of angular dependent PL measurements with optical simulation analysis, we developed the method to directly analyse a radiation pattern obtained in angular dependent PL measurements without the use of any optical simulations. The parameters used to evaluate the dipole orientation were *a*, *a*′, *a*″, *a*″′, *k*, *k*′, *k*″, *k*″′, *c*, *c*′, *c*″, *c*″′, *e*, *e*′, *e*″, and *e*″, which were determined by *d* and the substrate type. For practical use of this method, we have listed parameters for typical glass and fused silica substrates with a range of *d* values from 10 to 20 nm. The considered range of *d* is sufficiently small to be able to neglect the effects of *λ* in the visible range (400–700 nm), hence one can use this method without considering *λ*. For practical applications, a radiation pattern should be measured and the value of *n*_org_ determined. When we applied this calculation method to estimate *S*_calc_ for previously reported results from TADF emitters, the resultant *S*_calc_ values reproduced the reported values well with an error of less than 0.05. The error was consistent with our estimated error, which was evaluated by a comparison of *S*_calc_ with *S*_sim_ (Table 1). These results indicate that our calculation method is of potential use for practical estimations of dipole orientation.

This calculation method may not be suitable for all purposes, and if one is able to perform a full optical simulation, this would be the preferred way to analyse the data. However, this method shows potential for many diverse applications across research fields, in cases where the experimenter may not have experience in performing optical simulations. Dipole orientation is an important characteristic affecting photonic, electronic, excitonic, and optic properties of organic guest-host systems. This method may help researchers to rapidly calculated dipole orientations not only in OLED materials but many other binary molecular systems.

## Methods

### Optical Simulation

The radiation pattern was calculated by a photonic mode density simulation using a commercial software package (setfos 3.4, Fluxim Co.). In the calculation, the far-field emission intensity of the substrate material as a function of *θ* was simulated. *θ* was varied from 0° to 89° in steps of 1°. A light-emitting layer with a thickness *d* of 10, 15, or 20 nm was placed in-between the substrate and an air layer of infinite thicknesses. The refractive index of the substrate (*n*_sub_) was set to be either 1.524 for an ordinary glass or 1.460 for fused silica, while that for air (*n*_air_) was 1.000. The refractive index of the substrate of the organic light-emitting layer (*n*_org_) was varied from 1.600 to 2.200 in steps of 0.100. For practical purposes this refractive index value can be considered to be equivalent to that of the host matrix unless *n*_org_ is strongly influenced by the guest molecules. The extinction coefficients of all materials were assumed to be zero. The emission wavelength *λ* was varied in the range 400–700 nm in steps of 100 nm. An emitting dipole was placed at the centre of the light emitting layer, and the dipole orientation *Θ*_v_ was varied from 0 to 0.35 in steps of 0.05. Note that the simulation software uses *Θ*_v_ as the variable parameter, thus we translated *Θ*_v_ to *S*_sim_ for our analysis. The range of variation in *Θ*_v_ corresponded to *S*_sim_ of −0.50–0.05, meaning that the estimated dipole orientation completely covered the range from a horizontal orientation (*S*_sim_ = −0.5) to complete disorder (*S*_sim_ = 0). Although *S*_sim_ = 0.05 indicates a slight vertical orientation, previous studies have shown that such an orientation is feasible. We neglected the *S*_sim_ range from 0.05 to 1.00, because this degree of orientations can be considered outside of the range obtainable by conventional deposition methods.
